# Therapeutic Effect of pHLIP-mediated CEACAM6 Gene Silencing in Lung Adenocarcinoma

**DOI:** 10.1038/s41598-019-48104-5

**Published:** 2019-09-02

**Authors:** Seung-Myoung Son, Jieun Yun, Sung-Hoon Lee, Hye Sook Han, Young Hyun Lim, Chang Gok Woo, Ho-Chang Lee, Hyung Geun Song, Young-Mi Gu, Hyun-Jun Lee, Ok-Jun Lee

**Affiliations:** 1 Department of Pathology, Chungbuk National University Hospital, 776, 1Sunhwan-ro, Seowon-gu, Cheongju 28644 Republic of Korea; 2 Chungbuk National University College of Medicine, 1, Chungdae-ro, Seowon-gu, Cheongju 28644 Republic of Korea; 3 Department of Pharmaceutical Engineering, Cheongju University, 28, Andeokbeol-ro 104beon-gil, Cheongwon-gu, Cheongju 28503 Republic of Korea; 4 Department of Internal Medicine, Chungbuk National University Hospital, 776, 1Sunhwan-ro, Seowon-gu, Cheongju 28644 Republic of Korea; 5 Natural Medicine Research Center, Korea Research Institute of Bioscience and Biotechnology, 30, Yeongudanji-ro, Ochang-eup, Cheongwon-gu, Cheongju, Chungbuk 28116 Republic of Korea

**Keywords:** Targeted therapies, Cancer therapy

## Abstract

Carcinoembryonic antigen-related cell adhesion molecule 6 (CEACAM6) plays an important role in lung cancer progression. Here, we examined the therapeutic efficacy of *CEACAM6* gene silencing using an siRNA delivery platform targeting the acidic tumour microenvironment in a lung adenocarcinoma xenograft mouse model. An siRNA delivery vector was constructed by tethering the peptide nucleic acid form of an siRNA targeting *CEACAM6* (siCEACAM6) to a peptide with a low pH-induced transmembrane structure (pHLIP) to transport siRNAs across the plasma membrane. Specific binding of the pHLIP-siCEACAM6 conjugate to A549 lung adenocarcinoma cells at low pH was demonstrated by flow cytometry. A549 cells incubated with pHLIP-siCEACAM6 at an acidic pH showed downregulated expression of endogenous CEACAM6 protein and reduced cell viability. The *in vivo* tumour-suppressing effects of pHLIP-siCEACAM6 in lung adenocarcinoma were assessed in a xenograft model generated by injecting BALB/c nude mice with A549 cells. pHLIP-siCEACAM6 treatment alone resulted in tumour growth inhibition of up to 35.5%. When combined with cisplatin treatment, pHLIP-siCEACAM6 markedly enhanced tumour growth inhibition by up to 47%. In conclusion, the delivery of siCEACAM6 to lung adenocarcinoma using the pHLIP peptide has therapeutic potential as a unique cancer treatment approach.

## Introduction

Lung cancer is the leading cause of cancer-related mortality worldwide, with non-small cell lung cancer (NSCLC) accounting for 80–85% of lung cancer cases^1^. Despite improvements in early diagnosis and recent advances in the treatment of lung cancer, the prognosis of patients with NSCLC remains dismal^2^.

Carcinoembryonic antigen-related cell adhesion molecule 6 (CEACAM6) is a single-chain glycosyl phosphoinositol-anchored immunoglobulin-like glycoprotein that, along with other CEACAM family molecules, mediates homotypic and heterotypic cell–cell interactions through integrin receptors^3–5^. CEACAM6 is involved in cell adhesion, proliferation, migration, and invasion, as well as tumour cell metastasis^3,6^. It is overexpressed in a wide variety of carcinomas, including NSCLC and pancreatic, colon, breast, gastric, and hepatocellular carcinomas^7–17^. In NSCLC, lung adenocarcinomas express higher levels of CEACAM6 protein than other histologic subtypes. Although other CEACAM family proteins, such as CEACAM1 and CEACAM5, are known to be dysregulated in lung adenocarcinoma as well, CEACAM6 is more highly expressed than CEACAM5 and is associated with poor clinical outcomes among lung adenocarcinoma patients^3,7,13–15,18,19^. These data support a critical role of CEACAM6 in lung adenocarcinoma and suggest that it is an attractive therapeutic target. A recent study demonstrated the therapeutic potential of an anti-CEACAM6 monoclonal antibody by increasing the anoikis sensitivity in lung adenocarcinoma^20^; however, the use of RNA interference (RNAi)-based therapy to inhibit CEACAM6 in lung cancer has not been investigated.

RNAi is an endogenous cellular mechanism for regulating gene expression in which small-interfering RNAs (siRNAs) bind to the RNA-induced silencing complex to induce target mRNA cleavage and degradation through a catalytic process involving the Argonaute 2 endonuclease^21^. Since the first report of RNAi in 1998^22^, numerous studies have explored the use of RNAi-based approaches for therapeutic purposes^23^. siRNAs hold great promise as therapeutic agents in cancer because of their potential for silencing oncogenes^23,24^. While liposomes and nanoparticles provide certain advantages over viral-based systems for RNAi delivery, they exhibit low efficiency, as well as toxicity and rapid clearance from circulation^25^.

A peptide with a low pH-induced transmembrane structure (pHLIP) can be inserted into the cell membrane through the formation of an inducible transmembrane α-helix under acidic conditions and can translocate membrane-impermeable molecules into cells via a non-endocytic route^26,27^. Because of the acidic pH of the tumour microenvironment^28^, pHLIP can target a variety of solid tumours and avoid systemic clearance by the liver. Therefore, we decided to use pHLIP as a delivery vector for *CEACAM6*-targeting siRNA. Furthermore, we utilized a peptide nucleic acid (PNA) as a nucleic acid analogue of siRNA. PNAs are characterized by nucleobases joined by intramolecular amide bonds in which the phosphodiester backbone is replaced by a peptide-like backbone of repeated N-(2-aminoethyl)-glycine units. The lack of anionic phosphodiester groups increases the binding affinity for complementary nucleic acids. In addition, PNAs are resistant to nucleases and proteases and are stable across a wide range of pH values^29^. Cheng *et al*. utilized the charge-neutral properties of PNAs to tether PNA antisense oligomers to pHLIP, facilitating the intracellular delivery of the antisense oligomers^30^. We used the tumour-targeting properties of pHLIP and the potent binding affinity of the PNA to construct a tumour-targeted PNA delivery vector (pHLIP-PNA) by fusing pHLIP to the PNA form of an siRNA targeting *CEACAM6* (hereafter siCEACAM6). Here, we report the results of a concept study in a lung adenocarcinoma preclinical model that demonstrated the efficacy of *CEACAM6* silencing using pHLIP-mediated PNA siRNA delivery.

## Results

### The Cancer Genome Atlas (TCGA) data analysis

Analysis of a TCGA dataset of 515 lung adenocarcinoma patients using cBioPortal tools (http://www.cbioportal.org) showed CEACAM6 mRNA overexpression in 50 out of the 515 patients. Kaplan-Meier survival analysis showed that high levels of CEACAM6 mRNA expression were associated with poor overall survival in lung adenocarcinoma patients (*P* = 0.026) (Supplementary Fig. 1)^31^.

### Generation and assessment of the activity of pHLIP-PNA siCEACAM6

To generate the pHLIP-PNA siCEACAM6 conjugate, four siRNA sequences were screened for their efficacy in knocking down CEACAM6 expression. A549 lung adenocarcinoma cells were transfected with non-target siRNA, GAPDH siRNA, and four different siCEACAM6 sequences. All four sequences inhibited CEACAM6 mRNA expression to a similar level at 48 h after transfection at a concentration of 10 nM compared with the non-target siRNA (Supplementary Fig. 2a). siCEACAM6 sequence #1 was selected for tethering to pHLIP.

Additionally, since it is known that CEACAM1 and CEACAM5 are also dysregulated in lung cancer^3,13^, we assessed the effect of siCEACAM6 on the mRNA expression of CEACAM1 and CEACAM5 in A549 cells. The primers were designed from sequence regions unique to each member of CEACAM to obtain specific signals and avoid cross-reactivity with other members of this protein family. Our results showed that siCEACAM6 only inhibited the expression of endogenous CEACAM6 without affecting the mRNA expression of CEACAM1 or CEACAM5 in A549 cells (Supplementary Fig. 2b).

Next, the pHLIP-PNA siCEACAM6 construct was synthesized by conjugating the C-terminus of the PNA oligomer to pHLIP. A pHLIP-scr construct was generated as a control. The pHLIP-PNA was modified with a Single-isomer 5-carboxytetramethylrhodamine (TAMRA) label, which was attached to the PNA. The generation of pHLIP-PNA was verified by reversed-phase high performance liquid chromatography (RP-HPLC) and mass spectrometry (Supplementary Fig. 2c).

**Figure 1:**
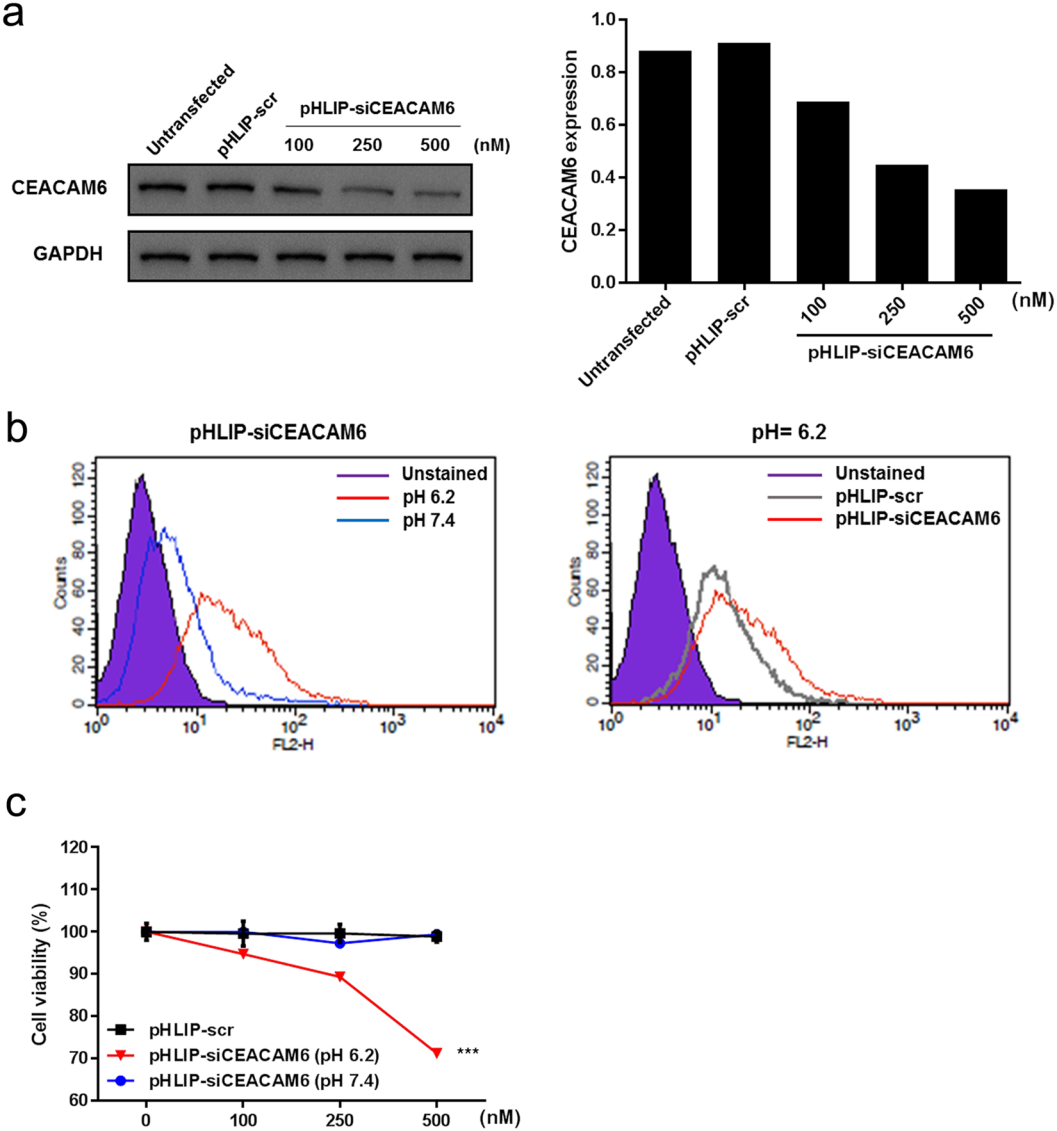
Assessment of the activity of pHLIP- siCEACAM6 in A549 cells. (**a**) Western blotting analysis of CEACAM6 protein levels in A549 cells incubated with pHLIP-siCEACAM6 at pH 6.2. (**b**) Flow cytometric analysis of A549 cells incubated with pHLIP-siCEACAM6 at neutral and acidic pH values. (**c**) Effects of CEACAM6 inhibition on A549 cell viability at neutral and acidic pH values. Data are shown as mean ± s.d.; ***P < 0.001.

To examine whether the pHLIP-PNA conjugate could deliver siCEACAM6 to A549 cells and downregulate CEACAM6, A549 cells were incubated with pHLIP-siCEACAM6 (100, 250, or 500 nM) or pHLIP-scr for 48 h at pH 6.2. Western blotting analyses showed that pHLIP-siCEACAM6 significantly downregulated the expression of endogenous CEACAM6 at low pH in a dose-dependent manner in A549 cells, confirming the intracellular delivery and inhibitory activity of siCEACAM6 (Fig. 1a). Flow cytometry was used to examine the A549 cell association at neutral and acidic pH values. A549 cells were incubated with pHLIP-siCEACAM6 or pHLIP-scr (500 nM) for 48 h at pH 7.4 or 6.2. Specific binding of pHLIP-siCEACAM6 to A549 cells was observed at a low pH. The attached PNA sequences did not affect the binding of pHLIP-PNA constructs (Fig. 1b). Assessment of cell viability at neutral and acidic pH values showed that inhibition of CEACAM6 by pHLIP-siCEACAM6 reduced A549 cell viability at pH 6.2 in a dose-dependent manner (Fig. 1c).

Additionally, we evaluated the effect of pHLIP-siCEACAM6 in two CEACAM6-epxressing colon cancer cell lines, HT29 and LoVo. As shown in A549 cells, pHLIP-siCEACAM6 downregulated CEACAM6 expression (Supplementary Fig. 3a,b) and reduced cell viability (Supplementary Fig. 3c,d) at low pH in a dose-dependent manner in both HT-29 and LoVo cells, respectively. Taken together, these data indicate that ligation of the pHLIP peptide to the PNA resulted in CEACAM6 mRNA targeting and CEACAM6 protein silencing by the delivered PNA siRNA.

### Therapeutic efficacy of siCEACAM6 in a lung cancer xenograft model

We first assessed the knockdown efficacy of delivered siCEACAM6 *in vivo*. For *in vivo* analysis, BALB/c nude mice were subcutaneously injected with 1.2 × 10^7^ A549 cells. Two weeks after tumour initiation, pHLIP-siCEACAM6 was injected into the mice via the tail vein with saline used as a negative control. To determine the optimal amount of pHLIP-siCEACAM6 for injection, two groups of mice (n = 5 mice/group) were injected with different doses (2 mg/kg and 4 mg/kg) twice per week for 3 weeks. Because cisplatin is the backbone of lung cancer treatment, cisplatin-treated mice served as the positive control. Mice received 2 mg/kg cisplatin intraperitoneally two or three times per week. After 3 weeks of treatment, all mice were sacrificed.

**Figure 2:**
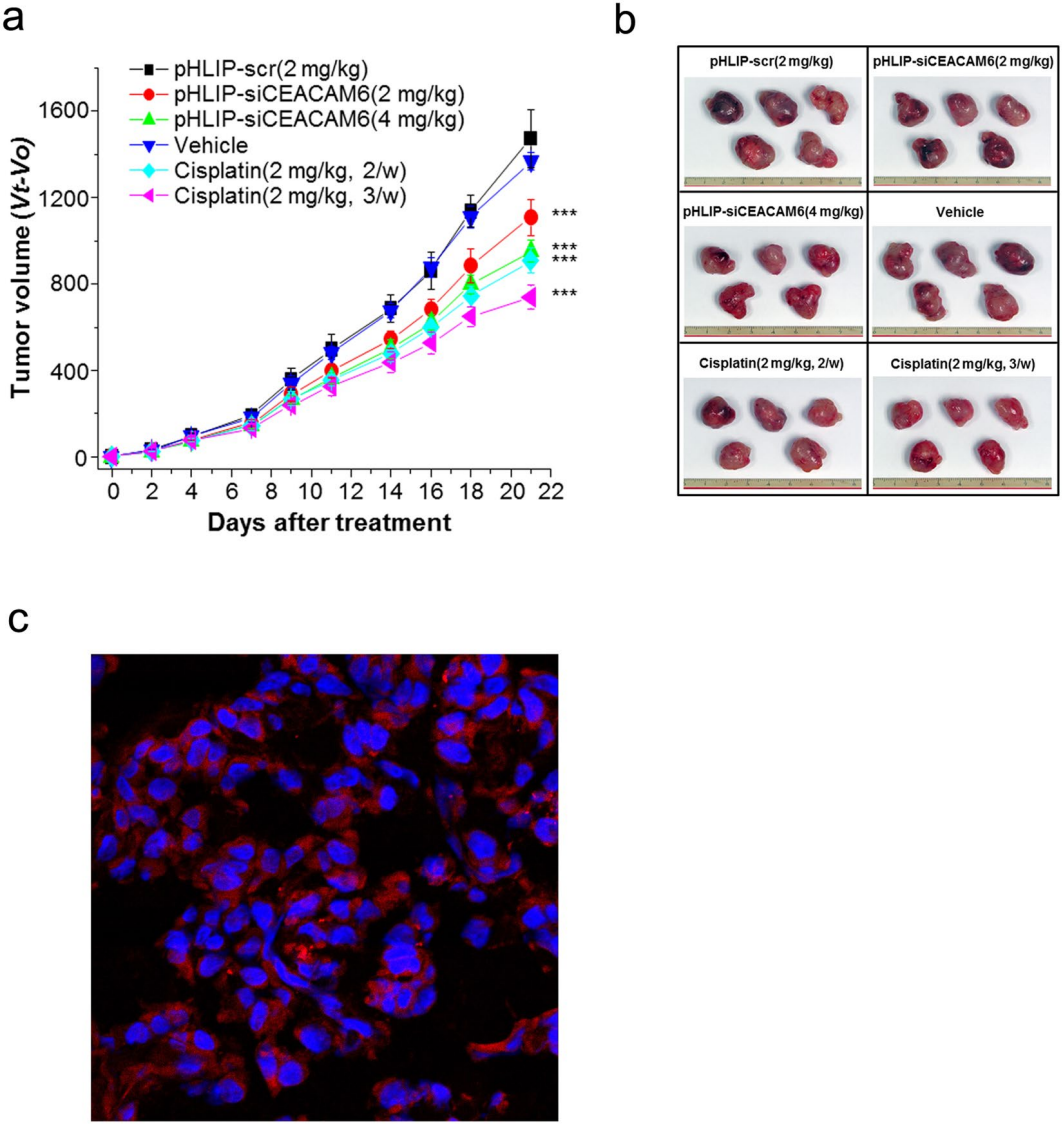
Targeted silencing of *CEACAM6* delays lung tumour progression in an A549 xenograft mouse model. (**a**) Nude mice bearing A549 tumours were injected intravenously with pHLIP-siCEACAM6 and cisplatin, and tumour volumes were measured thereafter (n = 5 mice/group). (**b**) Representative images of tumours from nude mice at 3 weeks after injection of pHLIP-scr, pHLIP-siCEACAM6, or cisplatin. (**c**) Confocal projections of A549 cells incubated with labelled pHLIP-siCEACAM6. Red, PNA-TAMRA; blue, nucleus. Data are shown as mean ± s.d.; ***P < 0.001.

**Figure 3:**
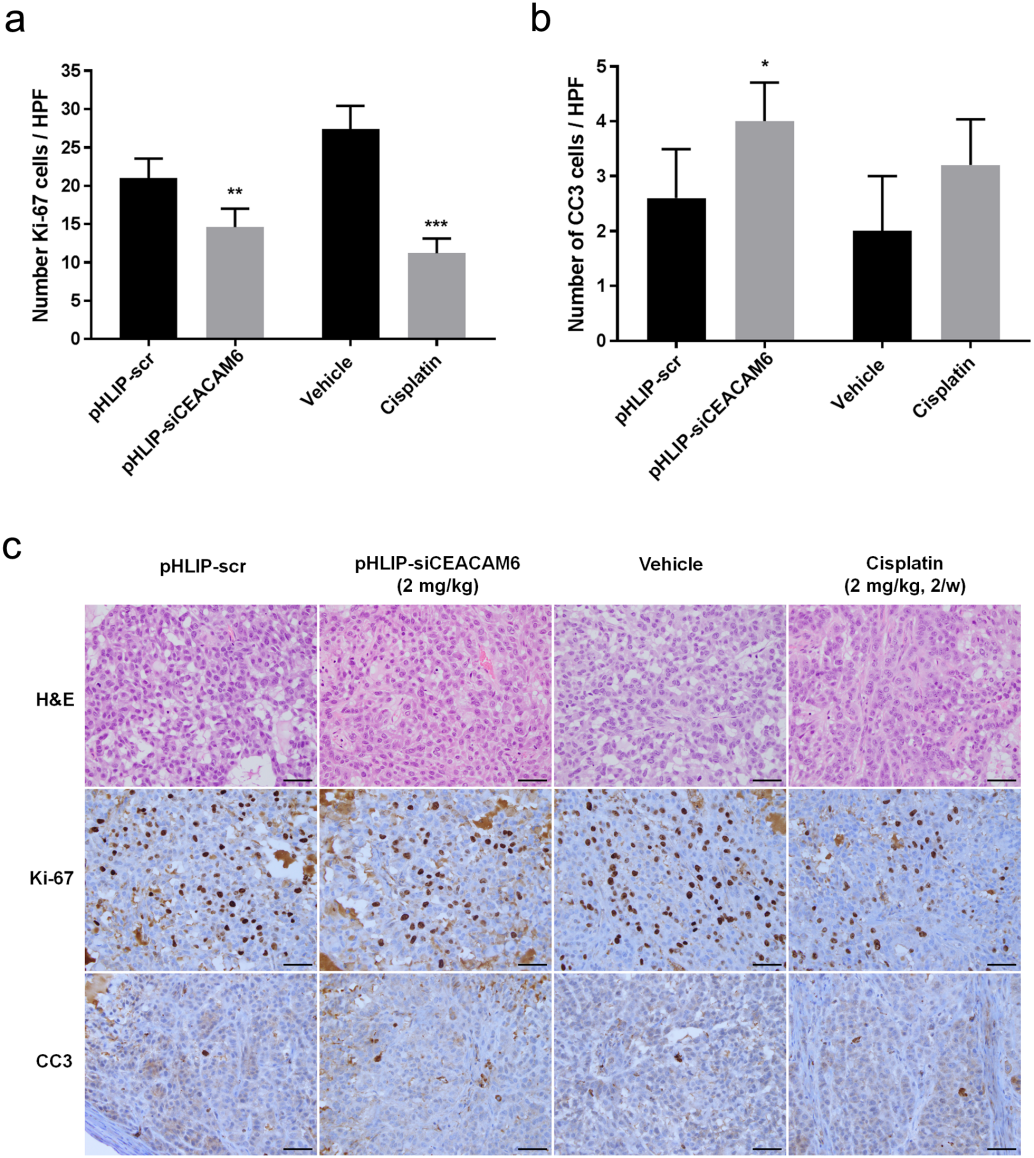
Quantification of dividing cells marked by Ki-67 staining (**a**) and apoptotic cells marked by CC3 staining (**b**) in treated tumours (n = 5 tumours/group). (**c**) Representative histology and immunohistochemical staining of lung tumours from mice treated with vehicle, pHLIP-scr, pHLIP-siCEACAM6, or cisplatin (original magnification × 400, scale bar = 50 µm). Data are shown as mean ± s.d.; *P < 0.05; **P < 0.01; ***P < 0.001.

To assess the effects of pHLIP-siCEACAM6 delivery on lung tumour development, tumour growth was measured every 2–3 days. pHLIP-siCEACAM6 treatment significantly suppressed tumour growth compared with that in control mice treated with pHLIP-scr (2 mg/kg pHLIP-siCEACAM6: 23.0% inhibition; 4 mg/kg pHLIP-siCEACAM6: 35.5% inhibition; all *P* < 0.001). A significant reduction in tumour growth was also observed in the cisplatin treatment group compared with that in vehicle-treated mice (cisplatin twice/week: 35.0%; cisplatin 3 times/week: 46.7%; both *P* < 0.001) (Fig. 2a,b). The ability of pHLIP-siCEACAM6 to deliver siCEACAM6 to lung adenocarcinomas *in vivo* was assessed by isolating tumour tissues for confocal imaging analysis. As pHLIP-siCEACAM6 was labelled with TAMRA, strong fluorescent signals were observed on the tumour cell surfaces of pHLIP-siCEACAM6-treated animals, indicating the efficient intracellular delivery of siCEACAM6 *in vivo* (Fig. 2c). Animals treated with siCEACAM6 or cisplatin exhibited no clinical signs of distress, body weight changes, or organ damage, including in the kidney, liver, and heart (Supplementary Fig. 4a,b). To investigate the mechanisms underlying tumour regression, lung tumours were harvested and stained for markers of proliferation and apoptosis. Proliferation levels were lower in pHLIP-siCEACAM6-treated tumours than in the pHLIP-scr treatment group (pHLIP-scr: 21.0%; pHLIP-siCEACAM6: 14.6%; *P* = 0.004). Cisplatin-treated tumours also showed a lower proliferation rate than vehicle-treated tumours (vehicle: 27.4%; cisplatin: 11.2%; *P* < 0.001). These data indicate that *in vivo* silencing of *CEACAM6* in tumours inhibits proliferation. Staining of cleaved caspase 3 (CC3), a marker of apoptosis, was higher in the pHLIP-siCEACAM6 treatment group than in pHLIP-scr tumours (*P* = 0.025) (Fig. 3a–c). These results suggest that delivery of exogenous siCEACAM6 suppressed lung tumour growth in a solid tumour xenograft mouse model.

### Therapeutic responses to concurrent treatment with siCEACAM6 and cisplatin

**Figure 4:**
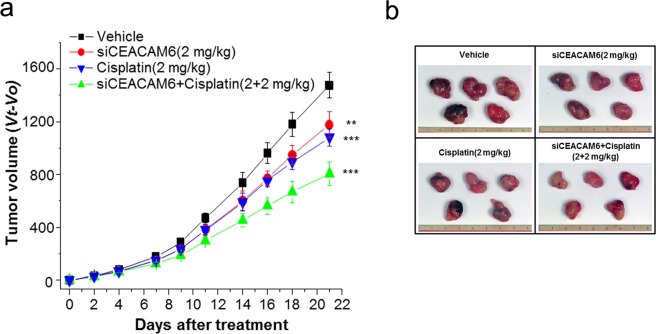
Concurrent treatment with siCEACAM6 and cisplatin improves therapeutic responses. (**a**) Nude mice bearing A549 tumours were injected intravenously with pHLIP-siCEACAM6 or cisplatin at the indicated times (arrows), and tumour volumes were measured thereafter (n = 5 mice/group). (**b**) Representative images of tumours from nude mice at 3 weeks after injection of pHLIP-scr, pHLIP-siCEACAM6, cisplatin, or pHLIP-siCEACAM6 + cisplatin. Data are shown as mean ± s.d.; **P < 0.01; ***P < 0.001.

Next, we assessed the antitumour effects of concurrent chemotherapy and *CEACAM6* targeting. Two weeks after subcutaneous injection of 1.2 × 10^7^ A549 cells, mice were randomly assigned to the following treatment groups (n = 5 mice/group): (i) vehicle, (ii) pHLIP-siCEACAM6, (iii) cisplatin, and (iv) pHLIP-siCEACAM6 + cisplatin. According to the *in vivo* results, a dose of 2 mg/kg pHLIP-siCEACAM6 was selected for combination treatment. Two weeks after cell line injection, pHLIP-siCEACAM6 was injected into the mice via the tail vein, and cisplatin was intraperitoneally injected twice per week for 3 weeks. Tumour volume was measured every 2–3 days. After 3 weeks of systemic therapy, mice in the pHLIP-siCEACAM6 treatment group exhibited a 21.8% reduction in tumour volume compared with that in vehicle-treated mice (*P* = 0.004). Cisplatin treatment reduced tumour volume by 29.9% (*P* < 0.001), whereas pHLIP-siCEACAM6 + cisplatin treatment significantly reduced tumour volume by 47% (*P* < 0.001) (Fig. 4a,b). Taken together, these results indicate that systemically delivered siCEACAM6 exerted significant therapeutic activity against primary tumours and that cytotoxic chemotherapy exhibited additive effects. None of the mice included in the therapeutic experiments experienced overt body weight loss or organ damage over the treatment period (Supplementary Fig. 5a,b).

**Figure 5:**
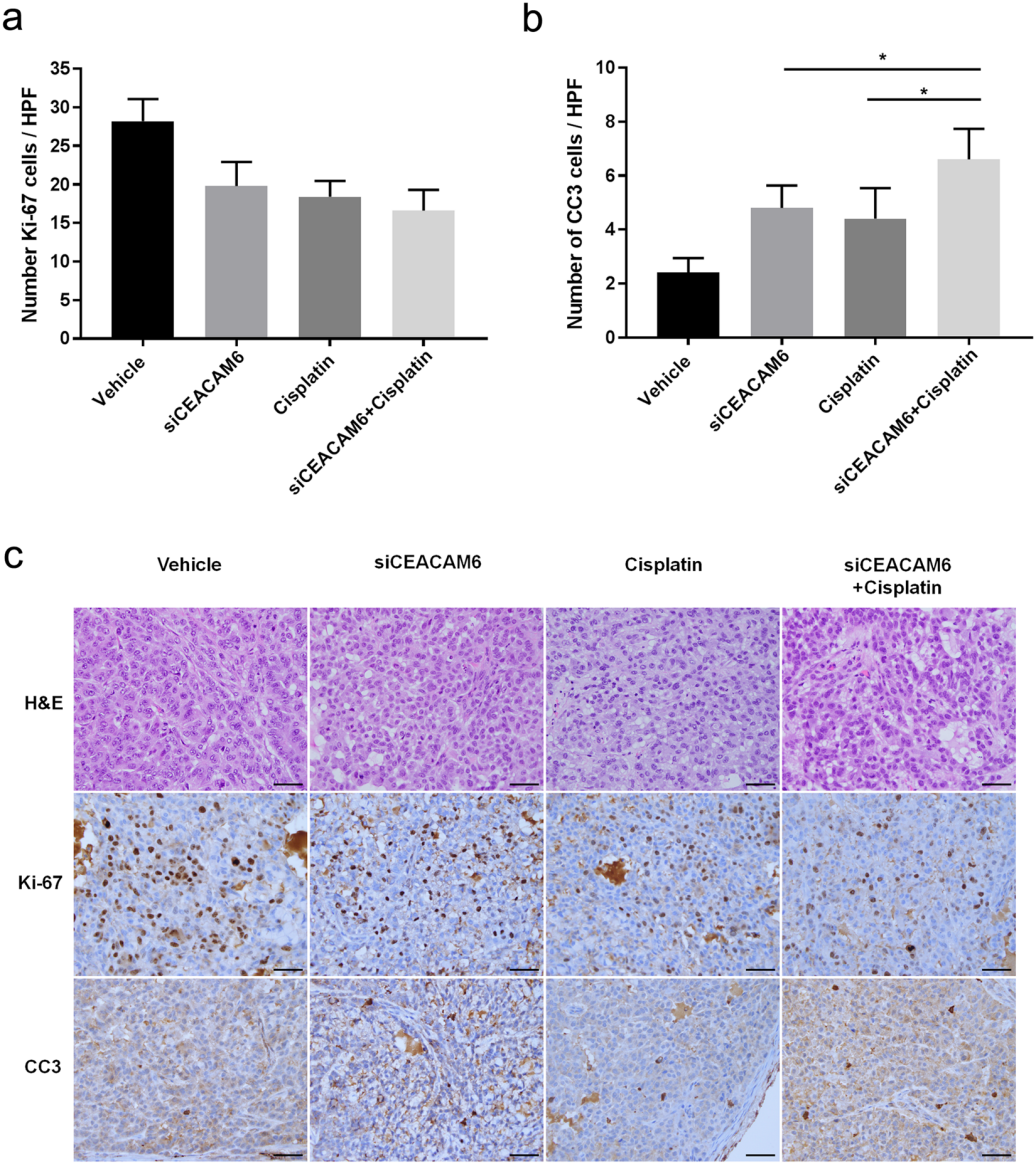
Quantification of dividing cells marked by Ki-67 staining (**a**) and apoptotic cells marked by CC3 staining (**b**) in treated tumours (n = 5 tumours/group). (**c**) Representative histology and immunohistochemical staining of lung tumours from mice treated with vehicle, pHLIP-siCEACAM6, cisplatin, or pHLIP-siCEACAM6 + cisplatin (original magnification × 400, scale bar = 50 µm). Data are shown as mean ± s.d.; *P < 0.05.

To further assess the biologic effects of systemic siCEACAM6 treatment, tumour samples were stained to assess proliferation and apoptosis. Cell proliferation in tumours treated with a combination of siCEACAM6 and cisplatin did not significantly differ from that in tumours treated with siCEACAM6 (*P* = 0.121) or cisplatin alone (*P* = 0.271) (siCEACAM6: 19.8%; cisplatin: 18.4%; pHLIP-siCEACAM6: 16.6%). However, combination-treated tumours showed higher numbers of CC3-positive cells than those treated with siCEACAM6 (*P* = 0.022) or cisplatin alone (*P* = 0.016) (Fig. 5a–c). Taken together, these results indicate that the combination of siCEACAM6 and cisplatin exhibited additive therapeutic effects, as determined by measurements of tumour volume and immunohistochemical staining of apoptosis markers.

## Discussion

In this study, we demonstrated that the pHLIP-siCEACAM6 conjugate is an effective molecule in treating lung adenocarcinoma that functions by targeting the acidic tumour microenvironment. Moreover, the addition of a chemotherapeutic drug to siCEACAM6 treatment elicited potent therapeutic responses.

A tumour-targeted siRNA delivery vector (pHLIP-siRNA) was constructed on the basis of the ability of pHLIP to transport siRNAs across the plasma membrane in the acidic tumour microenvironment. pHLIP can exist in three states: a water-soluble protein, a protein bound to the membrane surface, and a protein inserted into the lipid membrane as an α-helix^32–35^. At physiological pH, pHLIP is largely water-soluble, whereas at slightly acidic pH, pHLIP forms an α-helix capable of insertion into a lipid bilayer. Because of this characteristic, pHLIP is an attractive targeting moiety for selectively labelling and tracing acidic tissues *in vivo*^32^. As acidosis is a general property of the tumour microenvironment^28^, the pHLIP peptide localizes to tumours. We generated a pHLIP-siCEACAM6 conjugate by tethering the PNA form of siCEACAM6 to pHLIP, thereby translocating the PNA across the plasma membrane. The PNA and peptide are linked by a disulphide bond that is reduced in the cytosol; therefore, attachment of a PNA to the C-terminus of pHLIP promotes the intracellular delivery of the PNA.

Overexpression of CEACAM6 promotes cancer progression by inducing aberrant cell differentiation, anti-apoptosis, proliferation, and resistance to therapeutic agents^7^. Because a number of human adenocarcinomas express higher levels of CEACAM6 than other histologic subtypes, these results may have direct translational implications for the treatment of various human adenocarcinomas^7–17^. Several studies have explored the therapeutic targeting of CEACAM6 in various cancers using immunotherapeutic agents such as immunotoxin-based therapy^36^, monoclonal antibodies^6^, anti-CEACAM6 single chain variable fragment^37^, and antibody-drug conjugates^38^. In particular, a phase I/II trial of a protein conjugate named L-DOS47, consisting of a urease conjugated to a monoclonal antibody targeting CEACAM6, in NSCLC was reported. The urease enzyme of L-DOS47 converts urea into cytotoxic ammonia, and the ammonia increases the pH of the tumour microenvironment^39^.

Recently, the role of CEACAM6 in the inhibition of CD8+ T-cell responses in multiple myeloma was identified. Blocking CEACAM6 on the surface of myeloma cells restored T-cell reactivity against malignant plasma cells^40^. Wong *et al*.^41^ used the L-DOS47 immunoconjugate to augment the extracellular pH and restore the function of a human T lymphoblastoid cell line, promoting cell proliferation, IL-2 production, and cell surface PD-1 expression. The immune evasion property of CEACAM6 supports the use of therapeutic targeting of CEACAM6 as an immune checkpoint therapy.

The therapeutic use of siRNA against *CEACAM6* in pancreatic adenocarcinoma was studied by Duxbury *et al*.^42^ *CEACAM6* gene silencing resulted in tumour regression in a pancreatic adenocarcinoma xenograft model, including diminished tumour proliferation and improved survival. The present study is the first to report the therapeutic gene silencing of *CEACAM6* in lung adenocarcinoma. Moreover, the pHLIP vector was used to improve tumour-targeting activity and mediate the effective delivery of therapeutic small RNAs.

The present results using *CEACAM6*-targeting siRNA indicate that RNAi may effectively reduce CEACAM6 activity *in vitro* and induce antitumour effects in A549 human lung xenograft tumours. pHLIP-siCEACAM6 treatment significantly inhibited tumour growth with a superior antitumour response following a combination of RNA therapy and the chemotherapeutic drug cisplatin. The response to chemotherapy can be improved by specifically modulating genes involved in chemoresistance pathways. CEACAM6 overexpression results in chemoresistance through the Src-AKT pathway in various cancers, such as pancreatic adenocarcinoma^43,44^, cholangiocarcinoma^45^, and breast cancer^46^. Furthermore, CEACAM6 overexpression is associated with paclitaxel resistance in lung adenocarcinoma cells, and anti-CEACAM6 monoclonal antibody treatment enhances chemosensitivity *in vitro*^20^. In this study, siCEACAM6 delivery combined with cisplatin-based chemotherapy had an additive effect in suppressing tumour growth. The inhibition of CEACAM6 may regulate chemoresistance-related genes and increase the effect of cisplatin treatment. Therefore, RNA therapy may be combined with chemotherapy to improve the efficacy of existing cancer therapies.

No mice in our experiments exhibited any overt signs of toxicity. CEACAM6 is expressed on granulocytes and the epithelia of various organs^10^; however, because of the tumour-targeting property of pHLIP, siCEACAM6 delivery was limited to tumour tissues. Considering that CEACAM6 is also detected in the sera of cancer patients^47^, antibody-based therapies against CEACAM6 may be neutralized by the presence of circulating CEACAM6 in the serum before reaching cancer cells and producing effects. However, the pHLIP-siCEACAM6 developed in this study is directly delivered into the cytoplasm of tumour cells, minimizing the loss of therapeutic activity.

siRNA delivery as a therapeutic approach has many characteristics that make it desirable for further drug development. In first-in-human clinical trials, lipid nanoparticle-formulated siRNA delivery was found to be safe and well tolerated and, in some cases, elicited complete responses in metastatic cancer^48,49^. Future research will reveal whether treated tumours become resistant to small RNA-based therapies through mechanisms such as reduced nanoparticle uptake, increased nanoparticle exocytosis, and global downregulation of RNAi pathway function.

The present findings demonstrated that the pHLIP-siCEACAM6 conjugate is an effective antitumour molecule that targets the acidic tumour microenvironment and elicits potent antitumour responses when combined with chemotherapeutic drugs. The effective delivery of small RNAs to lung cancer cells using the pHLIP peptide is a unique approach to the use of small RNA therapies in cancer treatment.

## Methods

### Synthesis of PNA-pHLIP

PNA oligomers siCEACAM6 (TAMRA–ooo-GATCACAGTCTCTGGAAGT-ooo-Cys) and scrambled siRNA (scr; TAMRA–ooo-TGGTTTACATGTCGACTAA-ooo-Cys) were purchased from PANAGENE (Daejeon, South Korea). The PNA oligomer sequences including -ooo- (11-amino-3,6,9-trioxaundecanoic acid, a hydrophilic linker) and cysteine were generated by solid-phase synthesis using an automatic synthesizer with Bts PNA monomers, which are the proprietary PANAGENE building blocks for PNA oligomer synthesis. The oligomers were cleaved from the resin using m-cresol:TFA (1:4) cocktail solution. TAMRA was exclusively conjugated to the amino (N)-terminus of the PNA. The PNA oligomers were purified by RP-HPLC and characterized by matrix-assisted laser desorption/ionization–time of flight. The estimated melting temperatures of the siCEACAM6 and scrambled siRNA PNA oligomers were 79 °C and 75 °C, respectively.

To generate pHLIP-PNA constructs, the following pHLIP sequence was synthesized: AAEQNPIYWARYADWLFTTPLLLLDLALLVDADEGTCG. The C-terminus of each PNA oligomer was conjugated to pHLIP through a disulphide bond. To synthesize pHLIP-PNA constructs, pHLIP and PNA (peptide:PNA, 1.5:1) were reacted overnight in the dark in a mixture of DMF/Tris (pH 7.5). After conjugation, pHLIP-PNA was purified by RP-HPLC and characterized by matrix-assisted laser desorption/ionization–time of flight. The pHLIP-PNAs were freeze-dried after quantification by UV/VIS spectrophotometry at 260 nm using the following extinction coefficients: 13,700 M^−1^cm^−1^ (A), 6,600 M^−1^cm^−1^ (C), 11,700 M^−1^cm^−1^ (G), 8,800 M^−1^cm^−1^ (T), 200 M^−1^cm^**−**1^ (F, phenylalanine), and 32,300 M^−1^cm^−1^ (TAMRA).

### Cell culture

A549 human lung adenocarcinoma cell lines and HT-29 and LoVo human colon adenocarcinoma cell lines were purchased from the American Type Culture Collection. A549 and HT-29 were maintained in RPMI containing l-glutamine (2 mM), penicillin (100 U/ml), streptomycin (100 µg/ml), and 10% FBS at 37 °C in a 5% CO_2_ atmosphere. LoVo cells were maintained in F-12K containing penicillin (100 U/ml), streptomycin (100 µg/ml), and 10% FBS at 37 °C in a 5% CO_2_ atmosphere. For all pH-controlled cell culture experiments, cells were incubated with 10% FBS in the culture media buffered at pH 7.4 with HEPES or pH 6.2 with MES.

### Transient transfection with CEACAM6 siRNA

siRNA duplexes were purchased from Dharmacon Research, Inc. (Lafayette, CO, USA). To confirm the specificity of the inhibition, the control, non-target siRNA was used as the negative control. The siRNA sequences used for CEACAM6 siRNA experiments are as follows:

Negative control: UGGUUUACAUGUCGACUAA

Seq #1: GAUCACAGUCUCUGGAAGU,

Seq #2: GAACAUGGCUAAAUACAAU,

Seq #3: GAGGGUAACUUAACAGAGU,

Seq #4: CUACAUACUCCAACUGAAA.

A549 cells were transfected with 10 nM of siRNA duplexes using Lipofectamine RNAiMax (Thermo Fisher Scientific, Rockford, IL, USA) according to the manufacturer’s instructions. After 48 h, knockdown of the CEACAM6 gene was quantified by real-time RT-PCR.

### Quantitative real-time RT-PCR

Total RNA was isolated using the TRIzol reagent (Invitrogen, Carlsbad, CA, USA), and cDNA was synthesized using the iScript™ cDNA synthesis kit (Bio-Rad, Hercules, CA, USA) according to the manufacturer’s protocols. Quantitative real-time RT-PCR (qRT-PCR) was performed using iQ SYBR Green supermix (Bio-Rad). The primer sequences were as follows: GAPDH (forward, 5′-CCTGCACCACCAACTGCTTA-3′, reverse, 5′-GTCTTCTGGGTGGCAGTGAT-3′); CEACAM6 (forward, 5′-GACAGTTCCATGTATACCCG-3′, reverse, 5′-ACAGCATCCTTGTCCTCC-3′). CEACAM1 (forward, 5′- CCTATACCTGCCACGCCAAT-3′, reverse, 5′-TTGTGGAGCAGGTCAGGTTC-3′). CEACAM5 (forward, 5′-TTACCTTTCGGGAGCGAACC-3′, reverse, 5′-TTATTGCGGCCAGTAGCCAA-3′). Thermal cycling and fluorescence detection were performed using the S1000 thermal cycler real-time PCR system (Bio-Rad). Reactions were run in a CFX96 Real-Time PCR System (Bio-Rad) using the following thermal conditions: an initial denaturation step at 95 °C for 3 min, followed by 40 cycles of denaturation at 95 °C for 10 s and annealing/extension at 55 °C for 30 s. Gene expression levels were evaluated relative to GAPDH expression.

### Western blotting analysis

A549, HT-29, and LoVo cells were incubated with 10% FBS in in the culture media buffered at pH 7.4 with HEPES or pH 6.2 with MES and were treated with pHLIP-PNA suspended in reaction buffer for 48 h. Proteins were separated by sodium dodecyl sulphate–polyacrylamide gel electrophoresis and transferred to polyvinylidene fluoride membranes (Thermo Fisher Scientific). The membranes were blocked with 5% nonfat milk and then incubated with a mouse anti-CEACAM6 monoclonal antibody (9A6, Santa Cruz Biotechnology Inc., Dallas, TX, USA) or a mouse anti-GAPDH monoclonal antibody (6C5, Santa Cruz Biotechnology Inc.) as a loading control. The protein complexes were detected with enhanced chemiluminescence reagents (Thermo Fisher Scientific).

### Flow cytometry

A549 cells were incubated with 10% FBS in RPMI buffered at pH 7.4 with HEPES or pH 6.2 with MES and were treated with pHLIP-PNA suspended in reaction buffer for 48 h. After washing the cells to remove free pHLIP-PNA, they were resuspended in 500 μl PBS (pH 7.4) and analysed by flow cytometry (Becton Dickinson, San Jose, CA, USA).

### Cell viability

A Cell Counting Kit-8 (CCK-8) assay (Dojindo Laboratories, Kumamoto, Japan) was used to measure the effects of CEACAM6 inhibition on A549, HT-29, and LoVo cells. Cells (5 × 10^4^ cells/ml) were treated with pHLIP-siCEACAM6 at doses of 0, 100, 250, or 500 nM in 96-well plates for 48 h; pHLIP-scr served as the control. All treatments were performed at the indicated pH. After 48 h, the extent of cell growth was assessed using CCK-8 assay (Dojindo). CCK-8 solution (10 μl) was added to each well, followed by incubation for 2 h at 37 °C. The absorbance at 450 nm was determined by a multiplate reader (Lambda Bio-20; Beckman Coulter, Brea, CA, USA). Cell viability was expressed as a percentage of that in control cells.

### *In vivo* tumour xenograft experiments

All animal work was performed in accordance with a protocol approved by the Institutional Animal Care and Use Committee of the Korea Research Institute of Bioscience and Biotechnology. A549 cells were subcutaneously injected into the right flanks of 5-week-old BALB/c athymic nude mice (n = 5 mice/group; 1.2 × 10^7^ cells/mouse). Tumour growth was determined by measuring the length (L), width (W), and height (H) with callipers and using the formula V = (L × W × H) × 0.5. Upon tumour formation (approximately 44.8 mm^3^), mice were injected with pHLIP-PNA constructs intravenously and with cisplatin intraperitoneally as indicated.

### Confocal microscopy

Harvested tumour tissues were prepared for confocal microscopy by fresh freezing tumours in OCT before slicing into 2 μm sections. Tissue sections were fixed with cold acetone, rinsed with PBS, reacted with hydrogen peroxide to inactivate endogenous peroxidases, and washed with PBS. DAPI was added to stain cell nuclei. All washes were performed using PBS at pH 7.4 to remove surface-bound pHLIP. Images were acquired using a confocal microscope (Zeiss LSM 710 META, Jena, Germany) and processed using Zen software (version 8.0, Zeiss).

### Histology and other techniques

Mice were killed by carbon dioxide asphyxiation. Staining was performed in formalin-fixed, paraffin-embedded tumour sections (4-μm thickness). Fully automated immunostaining was achieved using a BenchMark XT autostainer (Ventana Medical Systems Inc., Tucson, AZ, USA). The following antibodies were used: monoclonal mouse anti-human CEACAM6 (9A6, 1:4,000; Santa Cruz Biotechnology Inc.), anti-CC3 (1:100, BioCare Medical, Pacheco, CA, USA), and anti-Ki-67 (1:100, VP-K452; Vector Laboratory, Burlington, Canada). The number of positive cells per tumour area was quantified.

### TCGA analysis with cBioPortal

A TCGA dataset (http://tcga-data.nci.nih.gov/) containing multidimensional genomic data combined with clinicopathological reports from 515 patients with lung adenocarcinoma was analysed using cBioPortal tools (http://www.cbioportal.org)^31^. RNA sequence data from lung adenocarcinoma patients from the TCGA were used for the analysis of CEACAM6 mRNA expression. For univariate survival analysis, Kaplan-Meier plots were created with the log-rank test using the overall survival data of lung adenocarcinoma patients from the TCGA.

### Statistical analysis

Two-way analysis of variance was performed for comparisons of cell viability data from three groups. For animal experiments, mouse and tumour weights for each group were compared using the Student’s *t*-test. The immunostaining results from each group were compared using the Student’s *t*-test. Survival curves were estimated using the Kaplan-Meier method and compared using the log-rank test. A *P* value ≤ 0.05 was deemed statistically significant. All statistical tests were two-sided and were performed using Prism 7 (GraphPad, La Jolla, CA, USA).

## Supplementary information

Supplementary information

## Data Availability

Data could be shared to this article.
