# Supplementary material for: Therapeutic Effect of pHLIP-mediated CEACAM6 Gene Silencing in Lung Adenocarcinoma
Source: Sci Rep. 2019 Sep 2;9:11607. doi: 10.1038/s41598-019-48104-5 (PMC6717735; doi:10.1038/s41598-019-48104-5)
Supplement: Supplementary file 1 — Supplementary information [file 41598_2019_48104_MOESM1_ESM.pdf]

Therapeutic Effect of pHLIP-mediated CEACAM6 Gene Silencing in Lung Adenocarcinoma

Seung-Myoung Son[1,2], Jieun Yun[3], Sung-Hoon Lee[3], Hye Sook Han[2,4], Young Hyun Lim[2], Chang Gok Woo[1,2], Ho-Chang Lee[1,2], Hyung Geun Song[1,2], Young-Mi Gu[5], Hyun-Jun Lee[5*], Ok-Jun Lee[1,2*]

[1] Department of Pathology, Chungbuk National University Hospital, 776, 1Sunhwan-ro, Seowon-gu, Cheongju, 28644, Republic of Korea

[2] Chungbuk National University College of Medicine, 1, Chungdae-ro, Seowon-gu, Cheongju, 28644, Republic of Korea

[3] Department of Pharmaceutical Engineering, Cheongju University, 28, Andeokbeol-ro 104beon-gil, Cheongwon-gu, Cheongju, 28503, Republic of Korea

[4] Department of Internal Medicine, Chungbuk National University Hospital, 776, 1Sunhwan-ro, Seowon-gu, Cheongju, 28644, Republic of Korea

[5] Natural Medicine Research Center, Korea Research Institute of Bioscience and Biotechnology, 30, Yeongudanji-ro, Ochang-eup, Cheongwon-gu, Cheongju, Chungbuk 28116, Republic of Korea

Supplementary Information

Supplementary Figure 1

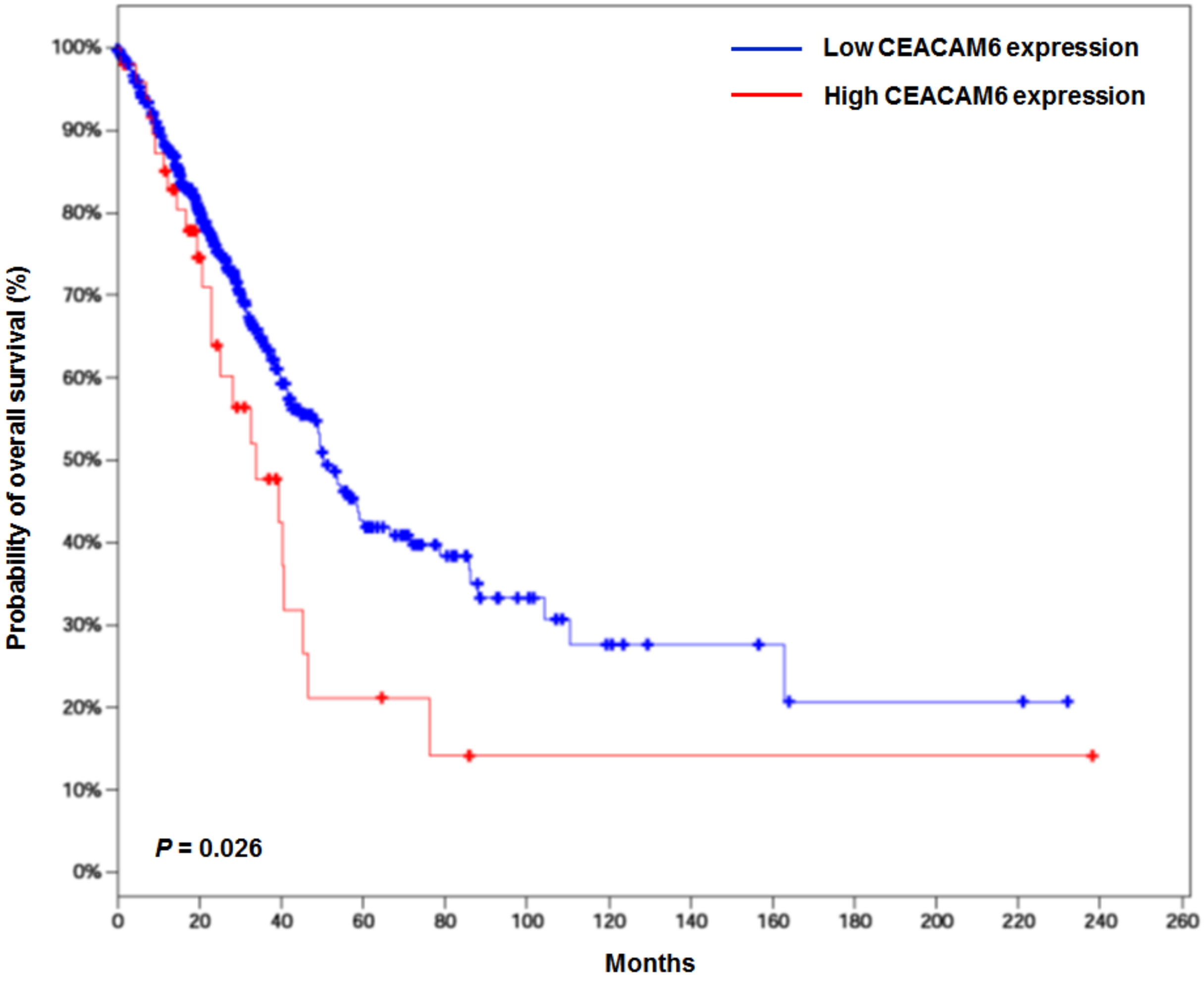

Supplementary Figure 1. Kaplan-Meier survival analysis of CEACAM6 mRNA expression in lung adenocarcinoma.

Supplementary Figure 2

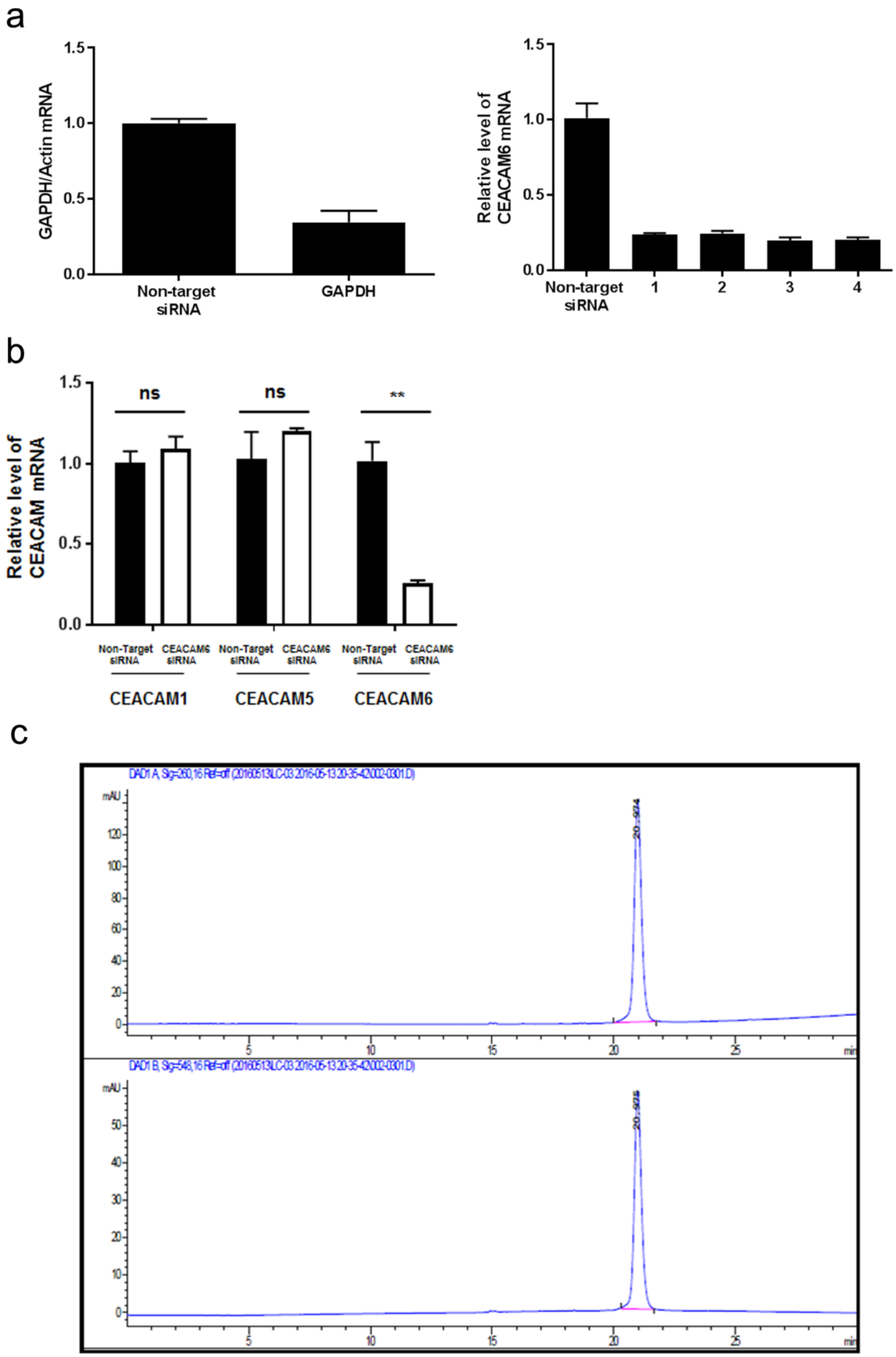

Supplementary Figure 2. Generation of pHLIP- siCEACAM6

(a) Quantitative RT-PCR of CEACAM6 mRNA expression in A549 cells transfected with non-target siRNA, GAPDH siRNA, or one of four different siCEACAM6 sequences. (b) Effects of siCEACAM6 on relative mRNA expression of CEACAM1, CEACAM5, and CEACAM6. (c) HPLC elution profiles of a reaction mixture of PNA (siCEACAM6) and pHLIP. Data are shown as mean ± s.d.; ns, not significant; $^{**}P < 0.01$.

Supplementary Figure 3

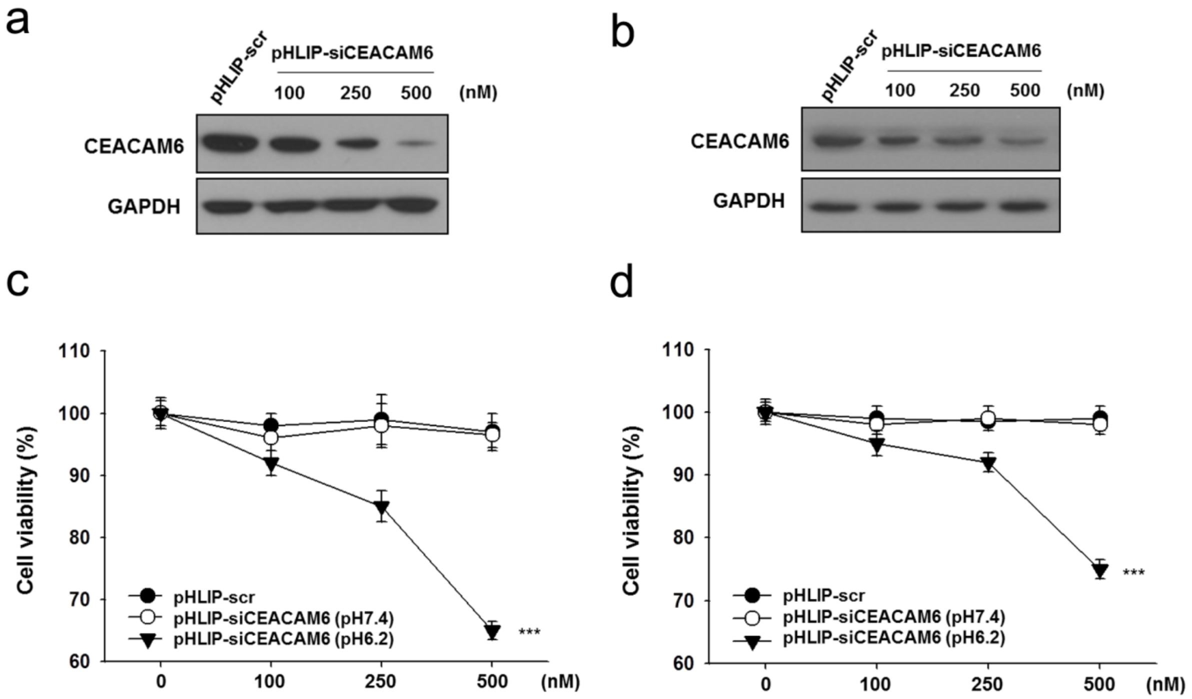

Supplementary Figure 3. Assessment of the activity of pHLIP- siCEACAM6 in HT-29 and LoVo colon adenocarcinoma cells

(a, b) Western blotting analysis of CEACAM6 protein levels in HT-29 cells (a) and LoVo cells (b) incubated with pHLIP-siCEACAM6 at pH 6.2. (c, d) Effects of CEACAM6 inhibition on viability of HT-29 cells (c) and LoVo cells (d) at neutral and acidic pH values. Data are shown as mean ± s.d.; $***P < 0.001$.

Supplementary Figure 4

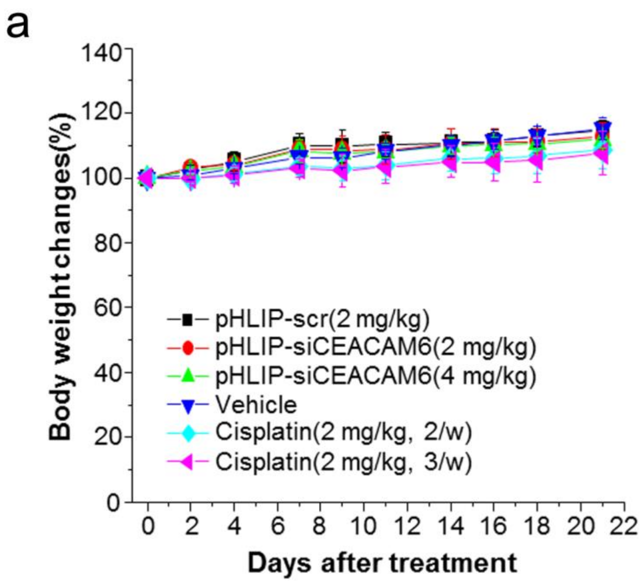

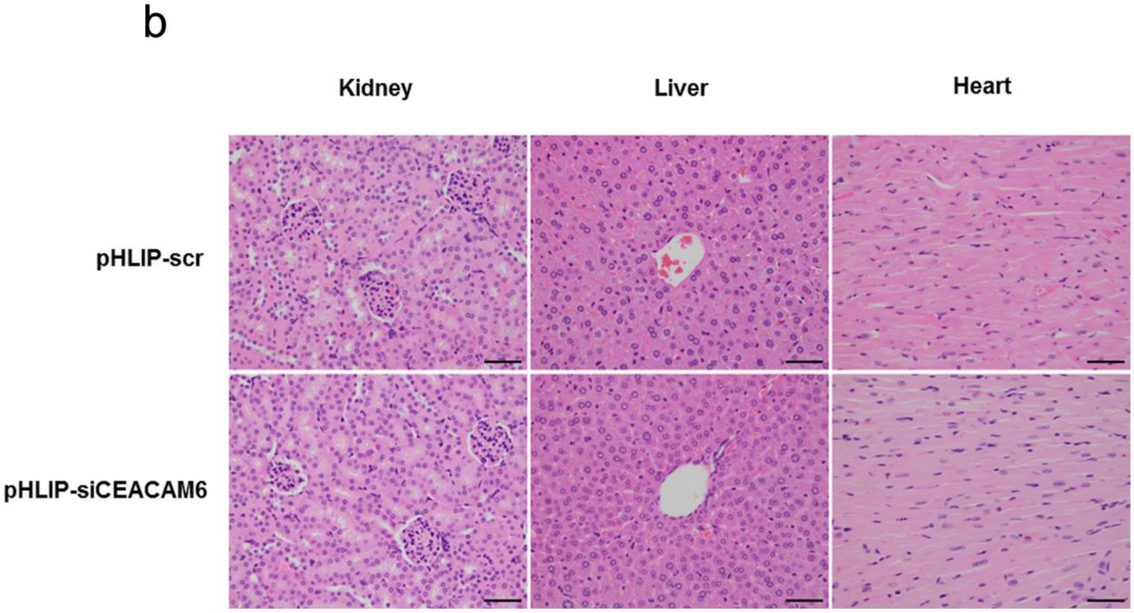

Supplementary Figure 4 Toxicity assessment of intravenously administered pHLIP-siCEACAM6

(a) Changes in body weight in experimental mice during the treatment period (n = 5 mice/group). Data are shown as mean ± s.d. (b) Representative histological analyses of the kidney, liver, and heart (original magnification × 400, scale bar = 50 µm) harvested from mice. All sections reveal an absence of microscopic changes associated with toxicity in pHLIP-siCEACAM6-treated animals (compare with normal sections in pHLIP-scr control).

Supplementary Figure 5

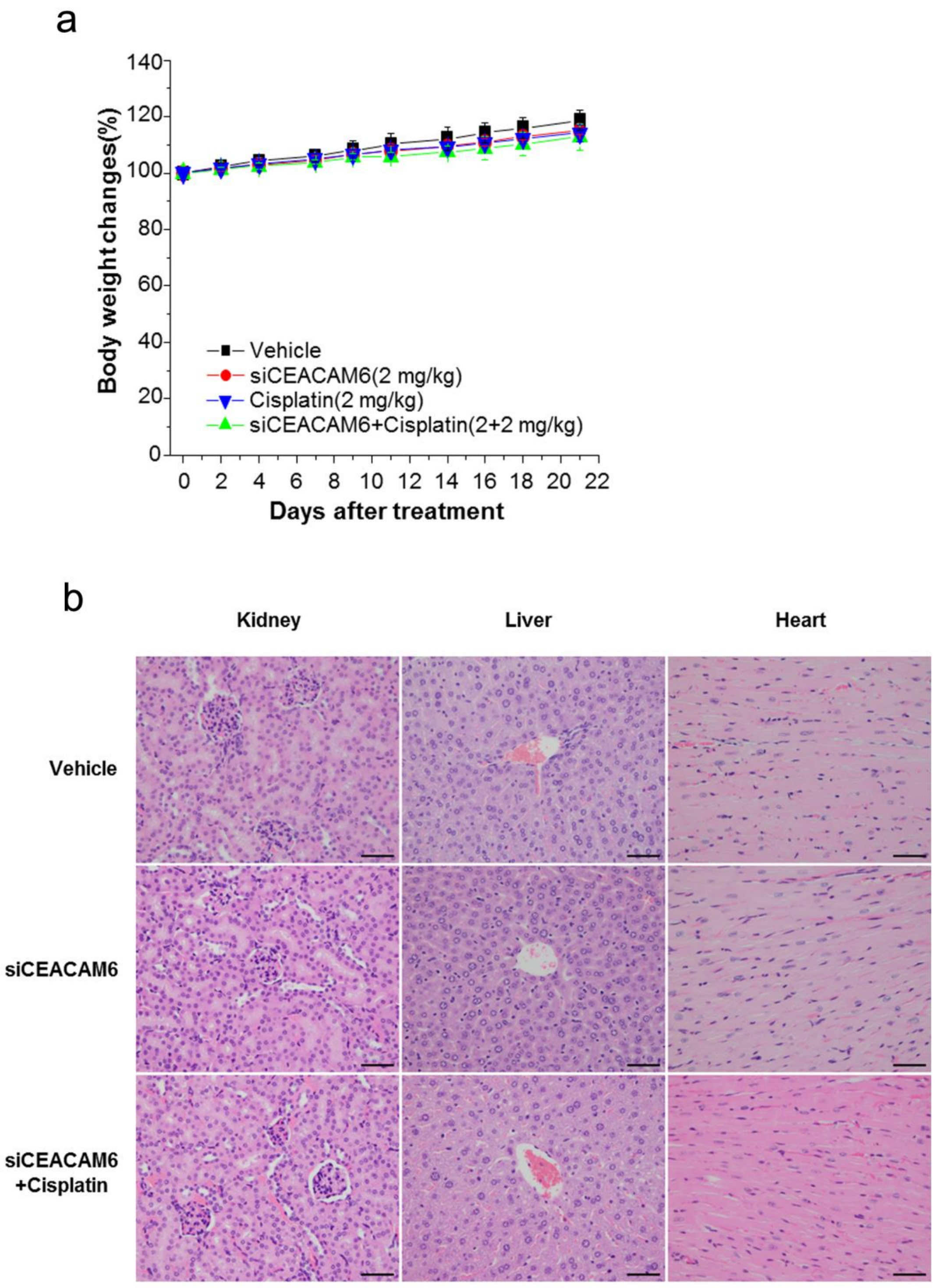

Supplementary Figure 5 Toxicity assessment of combination treatment with pHLIP-siCEACAM6 and cisplatin

(a) Changes in body weight in experimental mice during the treatment period (n = 5 mice/group). Data are shown as mean ± s.d. (b) Representative histological analysis of the kidney, liver, and heart (original magnification × 400, scale bar = 50 μm) harvested from mice. All sections reveal an absence of microscopic changes associated with toxicity in pHLIP-siCEACAM6-treated and pHLIP-siCEACAM6+cisplatin-treated animals (compare with normal sections in pHLIP-scr control).
